# The Role of Mitochondrial Mutations and Chronic Inflammation in Diabetes

**DOI:** 10.3390/ijms22136733

**Published:** 2021-06-23

**Authors:** Siarhei A. Dabravolski, Varvara A. Orekhova, Mirza S. Baig, Evgeny E. Bezsonov, Antonina V. Starodubova, Tatyana V. Popkova, Alexander N. Orekhov

**Affiliations:** 1 Department of Clinical Diagnostics, Vitebsk State Academy of Veterinary Medicine, 210026 Vitebsk, Belarus; siarhei.dabravolski@vsavm.by; 2 Laboratory of Angiopathology, Institute of General Pathology and Pathophysiology, 125315 Moscow, Russia; evgeny.bezsonov@gmail.com (E.E.B.); a.h.opexob@gmail.com (A.N.O.); 3 Department of Biosciences and Biomedical Engineering (BSBE), Indian Institute of Technology Indore (IITI), Simrol 456552, India; msb.iit@iiti.ac.in; 4 Institute of Human Morphology, 117418 Moscow, Russia; 5 Federal Research Centre for Nutrition, Biotechnology and Food Safety, 109240 Moscow, Russia; avs.ion@yandex.ru; 6 Therapy Faculty, Pirogov Russian National Research Medical University, 117997 Moscow, Russia; 7 V.A. Nasonova Institute of Rheumatology, 115522 Moscow, Russia; popkovatv@mail.ru

**Keywords:** diabetes, MIDD, mitochondria, mtDNA mutations, oxidative stress

## Abstract

Diabetes mellitus and related disorders significantly contribute to morbidity and mortality worldwide. Despite the advances in the current therapeutic methods, further development of anti-diabetic therapies is necessary. Mitochondrial dysfunction is known to be implicated in diabetes development. Moreover, specific types of mitochondrial diabetes have been discovered, such as MIDD (maternally inherited diabetes and deafness) and DAD (diabetes and Deafness). Hereditary mitochondrial disorders are caused by certain mutations in the mitochondrial DNA (mtDNA), which encodes for a substantial part of mitochondrial proteins and mitochondrial tRNA necessary for mitochondrial protein synthesis. Study of mtDNA mutations is challenging because the pathogenic phenotype associated with such mutations depends on the level of its heteroplasmy (proportion of mtDNA copies carrying the mutation) and can be tissue-specific. Nevertheless, modern sequencing methods have allowed describing and characterizing a number of mtDNA mutations associated with human disorders, and the list is constantly growing. In this review, we provide a list of mtDNA mutations associated with diabetes and related disorders and discuss the mechanisms of their involvement in the pathology development.

## 1. Introduction

Ageing is characterized by a gradual decline of the functioning of all body systems, with deterioration of cellular ability to proliferate and maintain homeostasis. Ageing-associated dysregulation of the immune system stimulates the production of pro-inflammatory cytokines even when typical inflammation triggers are not present. Such age-related chronic low-level systemic inflammation is often referred to as “inflammaging”, which had been described in several human disorders [1]. Previous studies have identified a range of molecular markers of a pro-inflammatory state, in addition to main cytokine markers (interleukin (IL)-1, IL-6, IL-8, IL-13 and IL-18, IL1 receptor (IL-1RN), tumor necrosis factor (TNF), interferon (IFN)-α, IFN-β, and tumor growth factor (TGF)-β). Recently, other proteins, such as C-reactive protein (CRP), TNFRSF members (1A and 1B) and serum amyloid A were added to the list [2]. Inflammaging is known for its involvement in human diseases, including cardiovascular disorders, cancer, diabetes, mental disorders, and neurodegeneration. Evidence of implication of inflammaging-associated processes in shorter life-span and premature death has also been reported [3,4].

Correct mitochondrial functioning is crucial for maintaining cellular metabolism and cell growth and proliferation. Not surprisingly, a range of human diseases and age-associated disorders were shown to be associated with mitochondrial dysfunction, including cancer, diabetes, cardiovascular diseases and atherosclerosis [5]. The existence of functional mitochondrial population within a cell, which responds to changing energy demands, depends on proper mitochondrial turnover. In this process, dysfunctional or excessive mitochondria can be cut into smaller fragments by mitochondrial fission and degraded through mitophagy, a specialized type of autophagy. Functional fragments can be joined together to form a new organelle through mitochondrial fusion process. Many pieces of evidence suggest that mitophagy is one of the key mechanisms for maintaining functional mitochondria population, and its deficiency can play an important role in various diseases [6]. The role of mitophagy in cellular physiology is vital, since it regulates cell differentiation and death. It also appears to be of great importance for the immune response processes [7]. Two major mitophagy pathways have been identified: PINK1-Parkin-dependent and PINK1-Parkin-independent. Under normal conditions, PINK1 protein is degraded, with Parkin (E3 ubiquitin ligase, required for degradation) located in the cytoplasm. However, in response to cellular damage or stress, mitochondrial PINK1 is autophosphorylated, upon which, Parkin is recruited to the mitochondria and mitophagy is induced. However, it was shown that in PINK1-deficient mice, Parkin can still relocate to the mitochondria and activate mitophagy, thus suggesting for the existence of some alternative mechanisms to compensate for PINK1 deficiency [8]. One of the main manifestations of impaired mitophagy and associated mitochondrial dysfunction is increased production of reactive oxygen species (ROS) that provoke low-grade and chronic inflammation [9].

Converging lines of research show that the efficiency of mitochondrial function gradually diminishes with age and is often associated with age-related pathologies [10]. One of the main reasons for this phenomenon is the accumulation of mutations in mitochondrial DNA (mtDNA), which leads to progressive deterioration of energy production and increase of ROS generation. The resulting ROS damage to the mtDNA leads to further mutagenesis. Mitochondrial DNA repair systems are not as reliable as nuclear ones, and mutated copies of mtDNA can accumulate with time, thus increasing the heteroplasmy level of harmful mutations. This forms a vicious cycle that contributes to the disease progression [11].

The role of mitochondrial malfunction, oxidative stress and inflammation in diabetes and its complications has been covered in previously published reviews [12,13]. In the current work, we provide an update of existing knowledge by summarizing the recent achievements in our understanding of the role of mitochondrial mutations in diabetes development and progression.

## 2. Inflammation in Diabetes

The term diabetes refers to a group of metabolic disorders usually defined by long-term elevated blood glucose level. Untreated or poorly managed diabetes is associated with complications affecting cardiovascular system, kidney, eyes, and nervous system. Currently, approximately 8.8% of world population (422 million) is estimated to have diabetes, and this proportion is predicted to increase with time. Type 2 diabetes (T2D) is more common and accounts for approximately 89% of diagnosed cases. Type 1 diabetes (T1D) has a prevalence of approximately 9% worldwide. T1D is characterized by insufficient insulin production due to beta cells failure as a result of autoimmune attack. The primary treatment for this type of diabetes is based on regular insulin injections. In T2D, cells fail to develop a proper response to insulin signaling, with insulin deficiency also possibly developing at later stages. Recommended preventive measures against T2D development include healthy lifestyle, adherence to diet, caloric restriction and regular training [14]. Gestational diabetes mellitus (GDM, also known as diabetes of pregnancy), occurs approximately in ~6% of pregnancies, and 90% of cases are resolved successfully after the delivery. Nevertheless, GDM patients have a higher risk of T2D development later in life [15]. Mitochondrial diabetes (MIDD or DAD) is a type of diabetes caused by a point mutation in m.A3243G mtDNA, affecting the gene encoding for the tRNA-Leu. This disease is characterized by maternal inheritance, progressive deafness and other symptoms, such as intestine malabsorption, cardiomyopathy, or kidney failure [16].

### 2.1. Type 1 Diabetes

T1D can be classified as autoimmune disease. In this condition, pancreatic β-cells are selectively attacked by the organism’s T-cells. In particular, CD8 and CD4 T-cells play a prominent role in β-cells destruction. These immune cells recognize a range of β-cells antigens: pro- and preproinsulin, insulinoma antigen, IGRP, GAD65, and islet amyloid polypeptide [17]. The exact mechanisms of triggering such autoimmune process remain largely unknown. Healthy people also have autoreactive T-cells, while autoantigen recognition does not occur [18]. It was suggested that micro-environmental factors, such as cytokines, oxidative stress and chemokines may alter antigen presentation and contribute to the recruitment of autoreactive T-cells to β-cells [19].

Normal functioning of pancreatic α- and β-cells is dependent on cytokine signaling. Under stress conditions, cytokine secretion is altered and is different in healthy people and T1D patients [20]. It is known that α-cells produce interleukin (IL)-1b, while both α- and β-cells produce IL-6, which contributes to the regulation of glucose homeostasis, rather than inflammation [20,21]. Other cytokines were found to be responsible for T1D pathogenesis and immune attack on β-cells, including interferon gamma (IFN-γ), CXCL10, IL-6, IL-17, IL-21, tumor necrosis factor (TNFα), and others, as well as cytokine receptors IL-4R, IL-6R, and IL-13R [22].

Several studies have confirmed the prominent role of the major histocompatibility complex (MHC) class I in T1D development. MHCI deficiency prevented the return of autoreactive T-cells to the islets [23]. At the same time, it was shown that IFN-α induces overexpression of MHCI [24], while IFN-γ that of MHCII [25]. These observations suggested the crucial role of the inflammation in the early steps of T1D development. Exposure of β-cells to pro-inflammatory cytokines resulted in overexpression of cytokines/chemokines (CXCL9, CXCL10 and CCL5) and MHCI, leading to endoplasmic reticulum (ER) stress and apoptosis [26]. Based on these results, it was suggested that pro-inflammatory cytokines cause a reduction of the proinsulin processing enzymes (PC1/3, PC2, and CPE) [27] and disrupt glucose-mediated insulin secretion, resulting in the accumulation of proinsulin in β-cells [28]. It was also suggested that mitochondria play the main role in the pro-inflammatory cytokines-mediated β-cells failure by restricting mitochondrial pyruvate oxidation capacity, increasing superoxide levels and inhibiting ATP synthesis [29].

### 2.2. Type 2 Diabetes

T2D is a chronic disease with oxidative, hormonal and epigenetic involvement, which is characterized by pathological hyperglycemia, which leads to further complications affecting the cardiovascular system, kidneys, retina and nervous system. Low-level chronic inflammation is one of the main causes of obesity-induced insulin resistance and T2D development, especially in patients of older age [30]. Pancreatic chronic inflammation in T2D, in combination with hyperglycemia and fatty infiltration, lead to increased apoptosis of β-cells, upregulated cytokine production, and reduced insulin biosynthesis [31]. Numerous studies in humans and animals have shown that adipose tissue acts as a main producer of pro-inflammatory cytokines, including TNFα, MCP-1, and IL-6. Therefore, obesity and inflammation appear to be interdependent in T2D development [32,33]. It was demonstrated that the abovementioned pro-inflammatory cytokines are tightly interconnected with the development of insulin resistance [34]. This effect may be direct, by interrupting the insulin signaling mechanism and down-regulating the insulin production [35], or indirect, by promoting macrophage infiltration and β-cells apoptosis [36]. Moreover, a general mechanism has been described, which involves hypothalamus functional deterioration mediated by IKK-β and NF-κB activation [37]. Patients with T2D present with accelerated aging processes, neuronal damage, and cognitive decline [38,39]. At the same time, it is well known that age-associated mitochondrial malfunction promotes insulin resistance in the elderly [40].

### 2.3. Maternally Inherited Diabetic Syndrome

A special subtype of maternally inherited diabetes, which is caused by a point mutation in the mitochondrial tRNA^(Leu)(UUR)^ gene, is referred to as Maternally Inherited Diabetes and Deafness (MIDD). The majority of MIDD cases are accompanied by neurosensory deafness. Unlike in other types of diabetes, in MIDD, a direct link is established between a mitochondrial DNA mutation, which is inherited along the maternal line, and the pathology. The mitochondrial mutation in question is m.A3243G, which affects the tRNA^(Leu)(UUR)^ gene. Because of that, this mutation has a broad impact, affecting the overall mitochondrial protein synthesis and impairing the stability of many mitochondrial proteins. The m.A3243G mutation is also known to cause mitochondrial myopathy, encephalopathy, lactic acidosis and stroke-like episodes (MELAS) syndrome, a condition associated with diabetes. The observed phenotype variety of m.A3243G mutation may be explained by its different impact on different organs and tissues. For instance, in the pancreas, m.A3243G mutation is associated with a low mass of β- and α-cells, which produce insulin and glucagon, respectively [41].

Usually, MIDD is not associated with insulin resistance, thus, does not require insulin treatment. The disease pathology is likely to be dependent on impaired energy production with inability of β-cells to properly and timely respond to glucose stimulation [42]. The discovery of the m.A3243G mutation, with its multifaceted physiological manifestations, was followed by intense studies that are summarized elsewhere [43].

## 3. The Role of Mitochondria in Diabetes

### 3.1. Mitochondrial Genome

**Figure 1:**
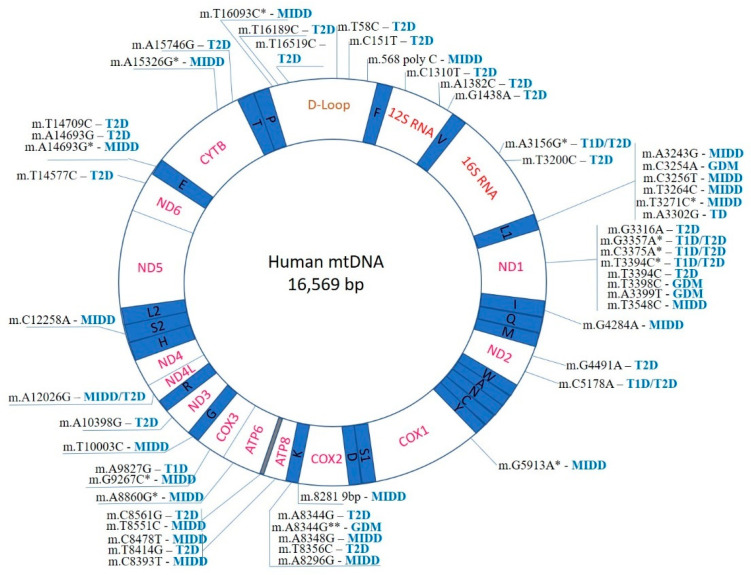
Mapping of known mtDNA mutations implicated in diabetes development. *- mutation identified in addition to m.3243G.

According to current understanding, mitochondria have emerged from endosymbiosis with proteobacteria, which explains the fact that they retain many bacterial features, including the genome organization. Human mtDNA is a double-stranded circular molecule, with the size of 16,569 bp. The two strands differ by nucleotide composition: the heavy strand (H) is enriched with guanine, while the light strand (L) is with cytosine. Mitochondria are partially autonomous, with a substantial part (but not all) of the mitochondrial proteins being encoded by the mtDNA and synthesized within the organelle. Importantly, 4 out of 5 main enzyme complexes responsible for oxidative phosphorylation (complexes I, III-IV) are assembled from 13 mtDNA-encoded proteins. Furthermore, mtDNA encodes 2 rRNAs (small 12S and large 16S) and 22 tRNA genes that carry out protein synthesis in the mitochondria (Figure 1). Coding regions are compactly packed within the mtDNA. The only non-coding region (D-loop) plays a regulatory role and contains H-strand transcription promotors and replication initiation sites for the entire mtDNA [44]. Such organization of the mitochondrial genome can explain why almost any mutation can potentially cause functional defects and lead to pathology development.

The number of mtDNA copies per cell can vary depending on the tissue, organ, and physiological condition. Mutations that are present in all mtDNA copies are called homoplasmic, while those that are carried by only a part of mtDNA copies are heteroplasmic. Depending on the level of heteroplasmy, mtDNA mutations can result in different effects at the molecular and cellular levels. Often, a specific phenotype develops when a certain threshold level mutation heteroplasmy is reached [6]. In many cases, mtDNA mutations are masked by functional wild-type copies. Phenotype alteration requires a significant level (usually, more than 70%) of heteroplasmy, which can also be tissue-specific [45].

### 3.2. The Search for Diabetes-Related Mitochondrial Mutations

Currently, the primary and simplest test for detecting diabetes is the oral glucose tolerance test. However, this approach cannot distinguish different types of diabetes due to the same cut-off values of the test [46]. Since diabetes is associated with metabolic changes in cells, it is logical to suspect that mitochondrial dysfunction can have a role in the pathology development. However, apart from relatively rare, maternally inherited syndromes, the causative relationship of mtDNA mutations with diabetes-related symptoms is difficult to establish. The dependence of pathologic manifestations on the heteroplasmy level, age-dependent accumulation and appearance of mtDNA mutations and the variety of effects of mtDNA mutations in different organs and tissues add complexity to this search. Nevertheless, the involvement of mitochondrial dysfunction associated with mtDNA mutations in the development of diabetes is being actively studied. It was possible to reveal a number of mutations that are likely to contribute to diabetes development and suggest potential mechanisms of their actions. Among the major known targets affected by mitochondrial dysfunction in diabetes are insulin signaling, synthesis and secretion pathways, and insulin sensitivity/resistance in the peripheral muscle [47,48].

Management of diabetes mellitus and assignment of insulin treatment depend on the observed symptoms and patient characteristics (body mass index, polyuria, polydipsia, the dynamic of weight gain/loss), and may require evaluation of β-cells status, presence of auto-antibodies and ketone bodies, and levels of C-peptide [49]. In the case of negative auto-antibodies test, C-peptide levels and absence of obvious symptoms, suitable T2D treatment should be assigned [50]. In some cases, genetic analysis should be considered. If maternal inheritance or gender-specific symptoms are suspected, it is necessary to examine the presence of mtDNA mutations. The approaches currently used for mtDNA mutation analysis include WES, mitoexome, mtDNA sequencing and WGS [51].

WES is mostly oriented at identification of different coding variants in a given genome. In particular, this method is useful in case of nuclear mutations. However, its efficiency and accuracy need to be improved [52]. Targeting WES specifically on mitochondrial exome (mitoexome) or small sets of genes resulted in improved resolution and allowed successful application of this method in clinical diagnostics to detect somatic and mitochondrial mutations [53]. WGS, as a more advanced technology, allows detecting the very low frequency of mtDNA mutations and has several advantages in speed and price [54].

Unlike NGS, which is a single strand sequencing technique, duplex sequencing methods can analyze both DNA strands, thus providing superior accuracy [55]. A recent invention called MitoRS is based on single-reaction primer-free amplification. This method is robust and sensitive and is well suited for analyzing a large set of samples with a low level of heteroplasmy [56].

Collection and analysis of samples for such genetic testing should be performed carefully. Due to the varying heteroplasmy levels in different tissues and organs, it is necessary to check several types of tissues. It is known that the heteroplasmy level for mtDNA mutations can be higher in post-mitotic tissues, such as muscle [57]. Moreover, the presence and heteroplasmy level of mtDNA mutations greatly varies between individuals. Additionally, mtDNA mutations may not only be maternally inherited but can also appear sporadically, making their analysis even more complicated, as was shown on monozygotic twins with and without diabetes [58].

Examination of liver samples demonstrated a significantly higher MAF and heteroplasmy level for the D-loop of the mitochondrial genome. Interestingly, the number of non-synonymous heteroplasmic mutations was age-dependent and subjected to positive selection [59]. Those data support the theory “survival of the slowest”, proposed in 1997 [60]. According to this theory, mitochondria accumulate mutations in the D-loop to suppress the respiratory function and reduce ROS production. Mutation-free mitochondria, in their turn, are ROS-damaged and eliminated from the cells, which leads to an increasing percentage of mutated mtDNA. By contrast, for hereditary mutations, the selection works against non-synonymous heteroplasmies [61]. mtDNA mutations inheritance patterns, natural polymorphism and heteroplasmy have already been studied for some neurogenerative diseases and several types of cancer [62], while for diabetes mellitus, such analysis remains a topic for future investigation.

### 3.3. Analysis of Pathogenic mtDNA Mutations in Diabetes Patients

Until the sequencing technologies became relatively widespread, “mtDNA diseases” were considered as uncommon conditions associated with severe symptoms (heart and cardiovascular system, neurodegenerative and age-related diseases and syndromes) and multisystem complications. Early studies of mtDNA mutations have suggested that numerous homoplasmic variations in mtDNA sequence were polymorphisms, while pathogenic mutations were heteroplasmic [63]. The cybrid (cytoplasmic hybrid) cell technology, developed in 1992 and used to study the MELAS syndrome, became the main tool for studying defects at the cellular level [64]. Detection of mtDNA mutations with cybrid cells technology requires a presence of particular clinical phenotype related to diabetes represented with high frequency. It is especially suitable to studying such mutations in a limited population (one family or group of close relatives). Wider population and mtDNA mutations with low frequencies require more sensitive and high throughput methods. Moreover, cybrid technology allows studying the possible mechanisms of mtDNA mutations’ pathological effects. To date, such mechanisms have been established for relatively few mtDNA mutations associated or identified in diabetes, most importantly, for the m.A3243G mutation in MIDD and MELAS. However, for numerous other mutations, causative role in diabetes development has not been proven yet (see Table S1).

In total, 54 mtDNA mutations related to different forms of diabetes have been identified so far, and this list is likely to grow (Figure 1). The majority of identified mutations are associated with T2D and MIDD diabetes (28 and 23, respectively). T1D and GDM have only a few associated mutations (6 and 4, respectively). Several hot-spot regions of the mitochondrial genome, where diabetes-associated mutations are especially frequent, can be identified. The D-loop region, a non-coding region controlling replication and translation of the mtDNA molecule, contains 6 mutations associated with T2D and MIDD (Figure 1). The region is known as the most variable within the mtDNA molecule, and several mtDNA alterations have been reported in human diseases, including different cancers. The exact mechanisms linking D-loop mutations to the formation of diabetic phenotype remain to be studied. These mutations are likely to affect the overall mitochondrial function through altered mtDNA replication and mtDNA depletion. The resulting ATP production deficiency affects the most sensitive cells and tissues contributing to the disease development. Depletion of mtDNA was demonstrated in some human cancers and other diseases. Age-dependent mtDNA depletion was observed in pancreatic β-cells, therefore providing for a link between reduced insulin production and accumulating mtDNA mutations in T2D [65].

A large group of mtDNA mutations associated with different types of diabetes are found in the regions responsible for mitochondrial translational system (rRNA and tRNA genes). The L1 and K regions contain 6 and 5 known mutations (associated with T2D, MIDD, or GDM), respectively (Figure 1). Mutations affecting these regions have also been reported in other human diseases, including ocular myopathy, myoclonic epilepsy and ragged red muscle fibers (MERRF) and cardiomyopathies. It is therefore interesting to compare the hot-spot of diabetes-related mtDNA mutations with hot-spots of other mitochondrial diseases [51].The effects of these mutations naturally affect mitochondrial protein synthesis resulting in overall impairment of mitochondrial function, reduced ATP production and cellular loss of function, which appears to be most prominent in cell types heavily dependent on energy production.

Mitochondrial protein-coding genes can also be affected by diabetes-associated mutations. As many as 7 mutations associated with different types of diabetes have been identified in the gene encoding for NADH–ubiquinone oxidoreductase subunit 1 (ND1), and several more in the genes encoding for other subunits (Figure 1). These mutations are likely to affect the redox balance of cells. Indeed, leukocytes from T2D diabetes patients carrying mutations in ND1 and ND2 genes were shown to have reduced ATP production in elevated generation of ROS [66].

The available data indicated that mtDNA mutations associated with various human diseases are most frequent in several hot-spot areas responsible for mtDNA replication, mitochondrial genes translation or synthesis of certain vital mitochondrial proteins. There is considerable overlap between the sets of mutations known to be associated with diabetes and with mitochondriopathies characterized by altered energy metabolism. Such overlap suggests the existence of common pathophysiological mechanisms causally linking mtDNA mutations with pathology development. However, a causal relationship needs to be proven for each mutation using such tools as cybrid and animal models. In particular, it is possible that age-associated phenotypic changes and accumulation of mtDNA mutations develop in parallel or are dependent on a common external factor, such as increased oxidative stress or exposure to certain toxins.

## 4. Study of Molecular Mechanisms of Diabetes-Associated mtDNA Mutations

### 4.1. Mouse Models

Mouse models provided a great contribution to our understanding the role of mitochondrial mutations in diabetes. ALR/Lt mouse strain has strong T1D resistance, while NOD/Lt strain is T1D-prone [67]. Crossing of these two strains helps in identifying SNPs in mt-Nd2 (MtA4738C) and mt-Co3 (MtA9827G) genes, related to T1D development. Maternally inherited ALR/Lt-specific SNPs in mt-Nd2 and mt-Co3 genes appear to be protective for β-cells against free radicals and oxidative stress [68].

As was shown in β-cells islet of p53^−/−^ mice, Parkin-mediated mitophagy is a crucial process in diabetes resistance. p53 directly interacts with Parkin, thus reducing mitophagy in β-cells and, subsequently, inhibiting insulin secretion under high glucose conditions [69]. Interestingly, p53 is known for its role as a tumor suppressor and regulator of redox and glucose metabolism [70], and participation in pancreatic β-cells mitophagy dysregulation provides a new line of evidence connecting cancer, inflammation and diabetes.

Small non-coding micro RNAs (miRNAs) are recently discovered gene expression regulators that can also influence the expression of mitochondrial genes. Studies in diabetic animal models have revealed that mitochondrial miRNAs are disturbed in diabetes, contributing to our understanding of the pathology mechanisms and suggesting for another possible way of therapeutic intervention. Animal studies have identified mitochondrial miRNA that stimulate the expression of mitochondrial target genes and may prove to be useful for study and treatment of diabetic cardiaomyopathy [71].

A natural m.G7778T polymorphism in the MT-ATP8 gene (Asp→Tyr substitution) was identified in the C57BL/6J-mtFVB/N (B6-mtFVB) mouse strain [72]. In pancreatic β-cells mitochondria, these mutations were associated with high ROS generation, impaired glucose sensitivity and insulin secretory function [72]. Another study demonstrated that this mitochondrial mutation was associated with higher susceptibility to autoimmune diseases (T1D, pancreatitis, nephritis and other) and impaired female fertility. Affected mitochondria were shown to have abnormal morphology and to generate more ROS and produce less ATP [73].

Recently, a low level of heteroplasmic naturally-occurring mutation (m.5172, in the OriL) was identified in a mouse strain (AKR/J (C57BL/6J-mtAKR/J; B6-mtAKR) on a C57BL/6J (B6) background). Normally mtDNA carries 60–70% of “11A” in the 5172 position, while “12A” heteroplasmy is linked to a shorter life span, lower mtDNA copy number, impaired glucose and lipid metabolism. Interestingly, OXPHOS function was not impacted by the heteroplasmy level [74].

The effects of pathogenic m.G13997A mutation in the MT-ND6 gene were studied in aged mito-mice ND6M mouse strain. Carriers of the m.G13997A mutation presented with ROS over-production, with further development of glucose intolerance and B-lymphoma. Those results suggest a connection between mitochondrial mutation, respiration defects and development of cancer and diabetes [75].

### 4.2. Preclinical and Clinical Studies

Mechanisms of mtDNA mutations effects contributing to diabetes development should be studied on key cell types involved in the disease pathogenesis, namely, pancreatic β-cells. Glucose uptake by β-cells is insulin-independent, therefore plasma glucose level defines the concentration of glucose that β-cells are able to metabolize. Metabolized glucose determines the ATP/ADP ratio within β-cells and regulates insulin release via Ca^2+^-dependent channel. Any mutation in mtDNA, leading to impaired protein synthesis and ATP production, causes impaired insulin production and/or secretion, glucose uptake and signalling. Moreover, affected mitochondria were shown to produce more toxic substances interrupting normal Ca^2+^-dependent signalling, and to be associated with hyperglycaemia [76]. Early studies have established the link between the respiratory chain function and glucose-dependent insulin secretion [77]. Recent research supports those results and confirms the susceptibility of mtDNA for ROS-mediated oxidation generated by the respiration chain [78].

Currently, there are no effective medicines to cure mitochondrial disorders, although numerous substances have been studied preclinically and are currently undergoing clinical trials. The majority of treatments rely on support for the respiratory chain functions (supply with cofactors like Q10, succinate, thiamine and others), replenishment of the antioxidant pool and correction of affected biochemical pathways with secondary metabolites [79].

While pharmacology so far could not provide reliable results, supportive lifestyle, regular exercise and training have been proven to improve respiratory chain efficiency, general physiological and mitochondrial functions. A better effect could be achieved when medications were combined with individualized diet (balanced, low calories, ketogenic) [80]. Multiple pieces of research have shown that a ketogenic diet has anti-oxidant and anti-inflammatory properties and improves muscle health and mitochondrial biogenesis [81]. The most promising results were obtained in terms of mitochondrial mutations. Results of the in vitro experiments suggest that a ketogenic diet could reduce the level of heteroplasmic mtDNA mutation by selective elimination, increase the total level of mtDNA and ameliorate the tissue [82,83]. Notwithstanding multiple promising outcomes, the nutritional approach in the treatment of diseases associated with mitochondrial mutations require further investigation.

### 4.3. Pharmacotherapy

Specific pharmacological treatment, prescribed in case of identification of diabetes-related mitochondrial mutations, is not available yet [84]. This can be explained by the variety of mtDNA mutations affecting different aspects of mitochondrial functioning, therefore requiring individualized treatment. Metformin, the most commonly used agent for treatment of T2D, is not recommended in the case of mitochondrial diabetes because it was shown to cause lactic acidosis [85]. The mechanism of metformin action is based on inhibition of mitochondrial glycerophosphate dehydrogenase in the liver, while lactic acidosis is associated mostly with accompanying diseases and syndromes of other organs, such as heart, liver, and kidney [86]. Application of metformin can be considered in some cases with extreme caution and regular monitoring of the lactate level [87].

The most effective and widely used treatments of the T2D include SGLT-2i, GLP-1 RA related structures and their derivatives. SGLT-2i inhibits the functioning of sodium-glucose transport protein 2 in the intestinal mucosa, leading to lower blood sugar and body weight, reduced non-fatal myocardial infarction and heart failure, and improvement of inflammation status, liver steatosis and uric acid concentrations [88]. GLP-1 Ras, or incretin mimetics used for T2D and obesity treatment, are analogues of GLP-1 and primarily act in the pancreas and liver. Side effects of such treatment include reduced hunger and lower stroke risk [89]. Since mitochondrial mutations are known to cause higher ROS production, they are especially dangerous for the cardiovascular system, where they can cause cardiomyopathy, atherosclerosis and hypertension. Diabetic patients with identified mitochondrial mutations are considered as a high-risk group for cardiovascular diseases. Therefore, the cardioprotective effects of SGLT-2i, GLP-1 RA and related substances have significant benefits in comparison to alternative treatments [90]. As was shown on a rat model, SGLT-2i (Empagliflozin) has a direct action at the mitochondrial level, improving mitochondrial functions, biogenesis and membrane potential. Most probably, SGLT-2i acts in the mitochondria through Tfam and NRF-1 signaling pathway [91]. Another SGLT-2i (Dapagliflozin) was shown to improve mitochondrial functions by increasing the levels of fusion–fission controlling proteins (Mfn1, Mfn2 and Fis1), preventing mitochondrial membrane depolarization, decreasing the level of oxidative stress damage and improving ADP/ATP ratio [92]. In addition, SGLT-2i application could be even more beneficial for older patients due to the known age-related dysregulation of SGLT-2 because of the impaired Ca^2+^ homeostasis between the mitochondria and sarcoplasmic reticulum [93].

Similarly, GLP-1 RAs are known to provide direct action on the mitochondria. It was shown on a rat model that liraglutide reduces chronic inflammation by lowering levels of TNFα and IL-1β in the hippocampus and markers of mitochondrial stress (BAX) [94] and promotes the production of anti-inflammatory molecules (arginase 1, IL-10 and TGFβ) [95]. Other studies demonstrated cardioprotective activities of liraglutide via activation of Parkin-mediated mitophagy and reduction of oxidative stress damage [96]. Due to the systemic effects of GLP1 RAs, some of them can be harmful, especially for elderly patients with comorbidities. In particular, suppressed appetite and decreased caloric intake can lead to weight loss and cause hypoglycemia. At the same time, gastrointestinal symptoms (diarrhea, nausea and vomiting) are very common [97,98].

The role of chronic inflammation and age-related decline of the mitochondrial efficiency, which is partially explained by accumulating mtDNA mutations, is currently well recognized. Nevertheless, the exact pathophysiology by which the mtDNA mutation lead to development of diabetes and related comorbidities remains to be studied in detail. Currently, the most probable reason is the extreme sensitivity of pancreatic β-cells to mitochondrial oxidative stress, resulting in quick defection of insulin secretion. However, other factors, such as hyperglycaemia, changes in lipid metabolism, violation of Ca^2+^ signaling, and other yet unknown factors cannot be excluded.

## 5. Conclusions

MtDNA mutations are common among DM patients. While the majority of mtDNA mutations are unique and tend to exist only in a particular population, some of them have prevalence over others. Heteroplasmic mtDNA mutations that are difficult to detect and to model in vitro represent the main challenge for researchers and clinicians. Mitochondrial diabetes-associated mutations identified so far tend to be located in mtDNA areas responsible for the chromosome replication, mitochondrial translational machinery, or certain mitochondrial genes encoding for important proteins, notably, NADH–ubiquinone oxidoreductase subunits. Available studies on cybrid, cellular, or animal models suggests that these mutations affect the mtDNA copy number and overall mitochondrial function, reduce energy production or result in elevated ROS generation. These processes are likely to be most deleterious in certain cell types, among which are pancreatic β-cells.

Thanks to the rapid development of sequencing technologies and accumulated knowledge about population-specific and unique mtDNA mutations, it is possible to predict the likelihood of disease development of progeny by analyzing genomes of family members, and thus, start treatment and disease-preventing intervention as soon as necessary. Investigation of every genome individually allows us to explore every mutation and define its effects on metabolism. In future, this would help to us to take a step forward in regard to personalized medicine and providing every patient with effective treatment.

## Data Availability

Not applicable.

## Supplementary Materials

The following are available online at https://www.mdpi.com/article/10.3390/ijms22136733/s1.
