# Supplementary material for: The Role of Mitochondrial Mutations and Chronic Inflammation in Diabetes
Source: Int J Mol Sci. 2021 Jun 23;22(13):6733. doi: 10.3390/ijms22136733 (PMC8268113; doi:10.3390/ijms22136733)
Supplement: Supplementary file 1 [file ijms-22-06733-s001.zip › ijms-1260786-SI.pdf]

International Journal of *Molecular Sciences*

MDPI

Supplementary Table S1

Supplementary Table S1 presents a summary of known mtDNA mutations related to the main forms of diabetes (T1D, T2D, MIDD and GDM). It combines data from case studies (one patient/family) and wide population analyses. All mentioned mutations were identified retrospectively, from patients with diagnosed diabetes (internationally recognized and clinically approved criteria, but still could vary depending on the local clinical practice).

Table S1. Diabetes mellitus-related mtDNA mutations.

Mutation	Gene	DM type	Patient population	Reference
m.T58C		T2D	Italian T2D patients	Cormio et al. 2009
m.C151T	OriH			
m.568 poly C length variation (up to 12 bp)	D-loop	MIDD	Turkish family case study	Janssen et al. 2006
m.8281 length variation, 2 9-bp repeats	tRNA^{Lys}			
m.C1310T	12S rRNA	T2D	Japanese T2D patients	Tawata et al. 1998
m.G1438A				
m.A12026G	ND4			
m.A1382C	MOTS-c/12S rRNA	T2D	Asian T2D patients	Zempo et al. 2021
m.A3156G*	16S rRNA	T1D, T2D	Japanese T1D, T2D and GDM patients	Ohkubo et al. 2001
m.G3357A*	ND1			
m.C3375A*				
m.T3394C*				
m.T3200C	16S rRNA	T2D	Chinese T2D patients	Yang et al. 2002
m.A3243G	tRNA^{Leu}	MIDD	Caucasian family case study	Van den Ouweland et al. 1992
m.C3256T	tRNA^{Leu}	MIDD	Japanese family case study	Hirai et al. 1998
m.T3264C	tRNA^{Leu}	MIDD	Japanese family case study	Suzuki et al. 1997
m.T3271C*	tRNA^{Leu}	MIDD	Taiwanese family case study	Chen et al. 2004
m.A3302G	tRNA^{Leu}	T2D	Han Chinese family case study	Ding et al. 2016
m.G3316A	ND1	T2D	Japanese patients	Odawara et al. 1996
m.T3394C	ND1	T2D	Chinese patients	Tang et al. 2006
m.A14693G	tRNA^{Glu}	T2D	Indonesian patients	Pranoto et al. 2005
m.C3254A	tRNA^{Leu}	GDM	Healthy subjects with no diabetes history and symptoms, Singapore	Chen et al. 2000
m.T3398C	ND1			
m.A3399T				
m.T3394C	ND1	T2D		Liao et al. 2008

m.G4491A	ND2		Han Chinese patients	
m.C5178A				
m.A10398G	ND3			
m.T16189C	mtDNA control region			
m.T16519C				
m.T3548C	ND1	MIDD	Brazilian patients	Crispim et al. 2008
m.A8348G	$tRNA^{Lys}$			
m.C8393T	ATP8			
m.C8478T				
m.T8551C	ATP6, ATP8			
m.A12026G	ND4			
m.G4284A	$tRNA^{Ile}$	MIDD	Italian patient sith multisystem progressing disorders	Corona et al. 2002
m.A4738C**	ND2	T1D	ALR/Lt, a mouse straing with strong resistance to T1D	Mathews et al. 2005
m.5172 11 and 12 as polymorphic locus**	OriL	T2D	AKR/J (C57BL/6J-mtAKR/J; B6-mtAKR) on a C57BL/6J (B6) mouse strain	Hirose et al. 2018
m.C5178A	ND2	T1D, T2D	Japanese population	Wang et al. 2001, Uchigata et al. 2002
m.G7778T natural polymorphism**	ATP8	T1D, T2D	C57BL/6J-mtFVB/N (B6-mtFVB) mouse strain	Weiss et al. 2012, Yu et al. 2009
m.A8296G	$tRNA^{Lys}$	MIDD	Japanese patients	Kameoka et al. 1998
m.A8344G	$tRNA^{Lys}$	T2D	Japanese family case	Suzuki et al. 1994
m.A8344G*	$tRNA^{Lys}$	GDM	Asian Indian population with no diabetes history	Khan et al. 2015
m.T8356C	$tRNA^{Lys}$	T2D	Coimbatore T2D patients	Vijaya Padma et al. 2010
m.T8414G	ATP8	T2D	Chinese Uyghur T2D patients	Jing et al. 2017
m.G2706A	16S RNA	T2D		
m.C8561G	ATP6, ATP8	T2D	Case study of one family	Kytövuori et al. 2016
m.A8860G*	ATP6	MIDD	Chinese family case study with MIDD and MELAS	Li et al. 2015a
m.A15326G*	CYB			
m.G9267C*	COX3	MIDD	Tunesian family case with MIDD	Tabebi et al. 2015
m.G5913A*	COX1			
m.A9827G**	COX3	T1D	ALR/Lt strain, NOD/Lt and NOD/LtDvs mouse sub-strains	Mathews et al. 2005
m.T10003C	$tRNA^{Gly}$	MIDD	Han Chinese family with MIDD	Li et al. 2015b, Liu et al. 2015

m.C12258A	tRNA^{Ser}	MIDD	British family case with MIDD	Lynn et al. 1998, Choo-Kang et al. 2002
m.G13997A**	ND6	T2D	Aged mito-mice ND6^{M} mouse strain	Hashizume et al. 2012
m.T14577C	ND6	T2D	Japanese family	Tawata et al. 2000
m.T14577C*	tRNA^{Glu}	MIDD	Han Chinese patients	Wang et al. 2016
m.A14693G*	tRNA^{Glu}	MIDD	Taiwan MELAS patient with DM mother and healthy siblings	Tzen et al. 2003
m.T14709C	tRNA^{Glu}	T2D	Patient with muscle weakness due to COX deficiency	Hao et al. 1995
m.A15746G	CYB	T2D	Taiwan T2D patients	Loo et al. 2014
m.T16093C*	Non-coding region	MIDD	Chinese patients with MIDD	Jiang et al. 2019
m.T16189C	mtDNA control region for replication and transcription	T2D	Asian patients	Park et al. 2008
			UK patients	Poulton et al. 2002
			Chinese patients	Weng et al. 2005
			Finnish patients	Soini et al. 2012

*- mutation identified in addition to m.3243G;
**- mouse model

References

Chen, Y., Liao, W.X., Roy, A.C., Loganath, A., and Ng, S.C. (2000). Mitochondrial gene mutations in gestational diabetes mellitus. Diabetes Research and Clinical Practice 48, 29–35.

Chen, Y.-N., Liou, C.-W., Huang, C.-C., Lin, T.-K., and Wei, Y.-H. (2004). Maternally inherited diabetes and deafness (MIDD) syndrome: a clinical and molecular genetic study of a Taiwanese family. Chang Gung Med J 27, 66–73.

Choo-Kang, A.T.W., Lynn, S., Taylor, G.A., Daly, M.E., Sihota, S.S., Wardell, T.M., Chinnery, P.F., Turnbull, D.M., and Walker, M. (2002). Defining the importance of mitochondrial gene defects in maternally inherited diabetes by sequencing the entire mitochondrial genome. Diabetes 51, 2317–2320.

Cormio, A., Milella, F., Marra, M., Pala, M., Lezza, A.M.S., Bonfigli, A.R., Franceschi, C., Cantatore, P., and Gadaleta, M.N. (2009). Variations at the H-strand replication origins of mitochondrial DNA and mitochondrial DNA content in the blood of type 2 diabetes patients. Biochimica et Biophysica Acta (BBA) - Bioenergetics 1787, 547–552.

Corona, P., Lamantea, E., Greco, M., Carrara, F., Agostino, A., Guidetti, D., Dotti, M.T., Mariotti, C., and Zeviani, M. (2002). Novel heteroplasmic mtDNA mutation in a family with heterogeneous clinical presentations. Ann Neurol. 51, 118–122.

Crispim, D., Estivalet, A.A.F., Roisenberg, I., Gross, J.L., and Canani, L.H. (2008). Prevalence of 15 mitochondrial DNA mutations among type 2 diabetic patients with or without clinical characteristics of maternally inherited diabetes and deafness. Arq Bras Endocrinol Metab 52, 1228–1235.

Ding, Y., Zhuo, G., and Zhang, C. (2016). The Mitochondrial tRNA Leu(UUR) A3302G Mutation may be Associated With Insulin Resistance in Woman With Polycystic Ovary Syndrome. Reprod Sci 23, 228–233.

Hao, H., Bonilla, E., Manfredi, G., DiMauro, S., and Moraes, C.T. (1995). Segregation patterns of a novel mutation in the mitochondrial tRNA glutamic acid gene associated with myopathy and diabetes mellitus. Am J Hum Genet 56, 1017–1025.

Hashizume, O., Shimizu, A., Yokota, M., Sugiyama, A., Nakada, K., Miyoshi, H., Itami, M., Ohira, M., Nagase, H., Takenaga, K., et al. (2012). Specific mitochondrial DNA mutation in mice regulates diabetes and lymphoma development. Proceedings of the National Academy of Sciences 109, 10528–10533.

Hirai, M., Suzuki, S., Onoda, M., Hinokio, Y., Ai, L., Hirai, A., Ohtomo, M., Komatsu, K., Kasuga, S., Satoh, Y., et al. (1996). Mitochondrial DNA 3394 Mutation in the NADH Dehydrogenase Subunit 1 Associated with Non-Insulin-Dependent Diabetes Mellitus. Biochemical and Biophysical Research Communications 219, 951–955.

Hirai, M., Suzuki, S., Onoda, M., Hinokio, Y., Hirai, A., Ohtomo, M., Chiba, M., Kasuga, S., Hirai, S., Satoh, Y., et al. (1998). Mitochondrial Deoxyribonucleic Acid 3256C-T Mutation in a Japanese Family with Noninsulin-Dependent Diabetes Mellitus 1. The Journal of Clinical Endocrinology & Metabolism 83, 992–994.

Hirose, M., Schilf, P., Gupta, Y., Zarse, K., Künstner, A., Fähnrich, A., Busch, H., Yin, J., Wright, M.N., Ziegler, A., et al. (2018). Low-level mitochondrial heteroplasmy modulates DNA replication, glucose metabolism and lifespan in mice. Sci Rep 8, 5872.

Janssen, G., Neu, A., 't Hart, L., van de Sande, C., and Antonie Maassen, J. (2006). Novel Mitochondrial DNA Length Variants and Genetic Instability in a Family with Diabetes and Deafness. Exp Clin Endocrinol Diabetes 114, 168–174.

Jiang, W., Li, R., Zhang, Y., Wang, P., Wu, T., Lin, J., Yu, J., and Gu, M. (2017). Mitochondrial DNA Mutations Associated with Type 2 Diabetes Mellitus in Chinese Uyghur Population. Sci Rep 7, 16989.

Jiang, Z., Zhang, Y., Yan, J., Li, F., Geng, X., Lu, H., Wei, X., Feng, Y., Wang, C., and Jia, W. (2019). De Novo Mutation of m.3243A>G together with m.16093T>C Associated with Atypical Clinical Features in a Pedigree with MIDD Syndrome. Journal of Diabetes Research 2019, 1–8.

Kameoka, K., Isotani, H., Tanaka, K., Azukari, K., Fujimura, Y., Shiota, Y., Sasaki, E., Majima, M., Furukawa, K., Haginomori, S., et al. (1998). Novel Mitochondrial DNA Mutation in tRNALys(8296A → G) Associated with Diabetes. Biochemical and Biophysical Research Communications 245, 523–527.

Khan, I.A., Shaik, N.A., Pasupuleti, N., Chava, S., Jahan, P., Hasan, Q., and Rao, P. (2015). Screening of mitochondrial mutations and insertion-deletion polymorphism in gestational diabetes mellitus in the Asian Indian population. Saudi J Biol Sci 22, 243–248.

Kytövuori, L., Lipponen, J., Rusanen, H., Komulainen, T., Martikainen, M.H., and Majamaa, K. (2016). A novel mutation m.8561C>G in MT-ATP6/8 causing a mitochondrial syndrome with ataxia, peripheral neuropathy, diabetes mellitus, and hypergonadotropic hypogonadism. J Neurol 263, 2188–2195.

Li, W., Zhang, W., Li, F., and Wang, C. (2015a). Mitochondrial genetic analysis in a Chinese family suffering from both mitochondrial encephalomyopathy with lactic acidosis and stroke-like episodes and diabetes. Int J Clin Exp Pathol 8, 7022–7027.

Li, W., Wen, C., Li, W., Wang, H., Guan, X., Zhang, W., Ye, W., and Lu, J. (2015b). The tRNAGly T10003C mutation in mitochondrial haplogroup M11b in a Chinese family with diabetes decreases the steady-state level of tRNAGly, increases aberrant reactive oxygen species production, and reduces mitochondrial membrane potential. Mol Cell Biochem 408, 171–179.

Liao, W.-Q., Pang, Y., Yu, C.-A., Wen, J.-Y., Zhang, Y.-G., and Li, X.-H. (2008). Novel Mutations of Mitochondrial DNA Associated with Type 2 Diabetes in Chinese Han Population. Tohoku J. Exp. Med. 215, 377–384.

Liu, H., Li, R., Li, W., Wang, M., Ji, J., Zheng, J., Mao, Z., Mo, J.Q., Jiang, P., Lu, J., et al. (2015). Maternally inherited diabetes is associated with a homoplasmic T10003C mutation in the mitochondrial tRNAGly gene. Mitochondrion 21, 49–57.

Loo, J.-H., Trejaut, J.A., Yen, J.-C., Chen, Z.-S., Ng, W.-M., Huang, C.-Y., Hsu, K.-N., Hung, K.-H., Hsiao, Y., Wei, Y.-H., et al. (2014). Mitochondrial DNA association study of type 2 diabetes with or without ischemic stroke in Taiwan. BMC Res Notes 7, 223.

Lynn, S., Wardell, T., Johnson, M.A., Chinnery, P.F., Daly, M.E., Walker, M., and Turnbull, D.M. (1998). Mitochondrial diabetes: investigation and identification of a novel mutation. Diabetes 47, 1800–1802.

Mathews, C.E., Leiter, E.H., Spirina, O., Bykhovskaya, Y., Gusdon, A.M., Ringquist, S., and Fischel-Ghodsian, N. (2005). mt-Nd2 Allele of the ALR/Lt mouse confers resistance against both chemically induced and autoimmune diabetes. Diabetologia 48, 261–267.

Odawara, M., Sasaki, K., and Yamashita, K. (1996). A G-to-A Substitution at Nucleotide Position 3316 in Mitochondrial DNA Is Associated with Japanese Non-Insulin-Dependent Diabetes Mellitus. Biochemical and Biophysical Research Communications 227, 147–151.

Ohkubo, K., Yamano, A., Nagashima, M., Mori, Y., Anzai, K., Akehi, Y., Nomiyama, R., Asano, T., Urae, A., and Ono, J. (2001). Mitochondrial Gene Mutations in the tRNALeu(UUR) Region and Diabetes: Prevalence and Clinical Phenotypes in Japan. Clinical Chemistry 47, 1641–1648.

van den Ouweland, J.M.W., Lemkes, H.H.P.J., Ruitenbeek, W., Sandkuijl, L.A., de Vijlder, M.F., Struyvenberg, P.A.A., van de Kamp, J.J.P., and Maassen, J.A. (1992). Mutation in mitochondrial tRNALeu(UUR) gene in a large pedigree with maternally transmitted type II diabetes mellitus and deafness. Nat Genet 1, 368–371.

Park, K.S., Chan, J.C., Chuang, L.-M., Suzuki, S., Araki, E., Nanjo, K., Ji, L., Ng, M., Nishi, M., Furuta, H., et al. (2008). A mitochondrial DNA variant at position 16189 is associated with type 2 diabetes mellitus in Asians. Diabetologia 51, 602–608.

Poulton, J. (2002). Type 2 diabetes is associated with a common mitochondrial variant: evidence from a population-based case-control study. Human Molecular Genetics 11, 1581–1583.

Poulton, J., Brown, M.S., Cooper, A., Marchington, D.R., and Phillips, D.I.W. (1998). A common mitochondrial DNA variant is associated with insulin resistance in adult life. Diabetologia 41, 54–58.

Pranoto, A. (2005). The Association of Mitochondrial DNA Mutation G3316a and T3394c with Diabetes Mellitus. p.

Soini, H.K., Moilanen, J.S., Finnila, S., and Majamaa, K. (2012). Mitochondrial DNA sequence variation in Finnish patients with matrilineal diabetes mellitus. BMC Res Notes 5, 350.

Suzuki, S., Hinokio, Y., Hirai, S., Onoda, M., Matsumoto, M., Ohtomo, M., Kawasaki, H., Satoh, Y., Akai, H., Abe, K., et al. (1994). Diabetes With Mitochondrial Gene tRNALYS Mutation. Diabetes Care 17, 1428–1432.

Suzuki, Y., Suzuki, S., Hinokio, Y., Chiba, M., Atsumi, Y., Hosokawa, K., Shimada, A., Asahina, T., and Matsuoka, K. (1997). Diabetes Associated With a Novel 3264 Mitochondrial tRNALeu(UUR) mutation. Diabetes Care 20, 1138–1140.

Tabebi, M., Mkaouar-Rebai, E., Mnif, M., Kallabi, F., Ben Mahmoud, A., Ben Saad, W., Charfi, N., Keskes-Ammar, L., Kamoun, H., Abid, M., et al. (2015). A novel mutation MT-COIII m.9267G>C and MT-COI m.5913G>A mutation in mitochondrial genes in a Tunisian family with maternally inherited diabetes and deafness (MIDD) associated with sever nephropathy. Biochemical and Biophysical Research Communications 459, 353–360.

Tang, D.-L., Zhou, X., Li, X., Zhao, L., and Liu, F. (2006). Variation of mitochondrial gene and the association with type 2 diabetes mellitus in a Chinese population. Diabetes Research and Clinical Practice 73, 77–82.

Tawata, M., Ohtaka, M., Iwase, E., Ikegishi, Y., Aida, K., and Onaya, T. (1998). New Mitochondrial DNA Homoplasmic Mutations Associated With Japanese Patients With Type 2 Diabetes. Diabetes 47, 276–277.

Tawata, M., Hayashi, J.I., Isobe, K., Ohkubo, E., Ohtaka, M., Chen, J., Aida, K., and Onaya, T. (2000). A new mitochondrial DNA mutation at 14577 T/C is probably a major pathogenic mutation for maternally inherited type 2 diabetes. Diabetes 49, 1269–1272.

Tzen, C.-Y., Thajeb, P., Wu, T.-Y., and Chen, S.-C. (2003). Melas with point mutations involving tRNALeu (A3243G) and tRNAGlu(A14693g). Muscle Nerve 28, 575–581.

Uchigata, Y., Okada, T., Gong, J.-S., Yamada, Y., Iwamoto, Y., and Tanaka, M. (2002). A Mitochondrial Genotype Associated With the Development of Autoimmune-Related Type 1 Diabetes. Diabetes Care 25, 2106–2106.

Vijaya Padma, V., Anitha, S., Santhini, E., Pradeepa, D., Tresa, D., Ganesan, P., Ishwarya, P., and Balakrishnan, R. (2010). Mitochondrial and nuclear gene mutations in the type 2 diabetes patients of Coimbatore population. Mol Cell Biochem 345, 223–229.

Wang, D., Taniyama, M., Suzuki, Y., Katagiri, T., and Ban, Y. (2001). Association of the mitochondrial DNA 5178A/C polymorphism with maternal inheritance and onset of type 2 diabetes in Japanese patients. Exp Clin Endocrinol Diabetes 109, 361–364.

Wang, M., Liu, H., Zheng, J., Chen, B., Zhou, M., Fan, W., Wang, H., Liang, X., Zhou, X., Eriani, G., et al. (2016). A Deafness- and Diabetes-associated tRNA Mutation Causes Deficient Pseudouridinylation at Position 55 in tRNAGlu and Mitochondrial Dysfunction. Journal of Biological Chemistry 291, 21029–21041.

Weiss, H., Wester-Rosenloef, L., Koch, C., Koch, F., Baltrusch, S., Tiedge, M., and Ibrahim, S. (2012). The Mitochondrial Atp8 Mutation Induces Mitochondrial ROS Generation, Secretory Dysfunction, and β-Cell Mass Adaptation in Conplastic B6-mtFVB Mice. Endocrinology 153, 4666–4676.

Weng, S.-W., Liou, C.-W., Lin, T.-K., Wei, Y.-H., Lee, C.-F., Eng, H.-L., Chen, S.-D., Liu, R.-T., Chen, J.-F., Chen, I.-Y., et al. (2005). Association of Mitochondrial Deoxyribonucleic Acid 16189 Variant (T→C Transition) with Metabolic Syndrome in Chinese Adults. The Journal of Clinical Endocrinology & Metabolism 90, 5037–5040.

Yang, T., Lam, C.-W., Tsang, M.-W., Tong, S.-F., Kam, G.Y.W., Chan, L.Y.S., Poon, P.M.K., Wu, X., and Pang, C.-P. (2002). Novel mitochondrial 16S rRNA mutation, 3200T-->C, associated with adult-onset type 2 diabetes. Chin Med J (Engl) 115, 753–758.

Yu, X., Wester-Rosenlöf, L., Gimsa, U., Holzhueter, S.-A., Marques, A., Jonas, L., Hagenow, K., Kunz, M., Nizze, H., Tiedge, M., et al. (2009). The mtDNA nt7778 G/T polymorphism affects autoimmune diseases and reproductive performance in the mouse. Human Molecular Genetics 18, 4689–4698.

Zempo, H., Kim, S.-J., Fuku, N., Nishida, Y., Higaki, Y., Wan, J., Yen, K., Miller, B., Vicinanza, R., Miyamoto-Mikami, E., et al. (2021). A pro-diabetogenic mtDNA polymorphism in the mitochondrial-derived peptide, MOTS-c. Aging.
